# Community social capital and the health-related quality of life among empty-nest elderly in western China: moderating effect of living arrangements

**DOI:** 10.1186/s12888-022-04310-6

**Published:** 2022-11-04

**Authors:** Huan Zhu, Lei He, Jiayi Peng, Xingyue LI, Bo Gao, Huan Zhou, Yang Wan

**Affiliations:** 1 West China School of Public Health and West China Fourth Hospital, Sichuan University, 610041 Sichuan, China; 2 West China-PUMC C.C. Chen Institute of Health, Sichuan University, 610041 Sichuan, China

**Keywords:** Empty-nest elderly, Community, Social capital, Health-related quality of life

## Abstract

**Background:**

At present, the empty nest phenomenon is becoming more and more serious in the world, especially in China, and its health problems deserve attention. Therefore, the purpose of this study is to explore the impact of community social capital on the health-related quality of life of empty-nest elderly.

**Methods:**

The study used data collected from a survey study conducted between 2018 and 2019 in Sichuan province, China, with 638 empty-nest elderly meeting our criteria. SF-12 scale and self-made social capital scale were used to evaluate the health-related quality of life and community social capital of empty-nest elderly. Through descriptive statistical analysis, bivariate analysis and hierarchical multiple regression model, this study analyzes the relationship between community social capital and health-related quality of life of empty-nest elderly. Considering the role of living arrangements, this study further uses the simple effect analysis method to explore the moderating role of living arrangements.

**Results:**

After controlling the relevant variables, the cognitive social capital (CSC) of empty-nest elderly had a positive relationship with the physical health summary (PCS) (*β* = 0.188, *p*＜0.001) and mental health summary (MCS) (*β* = 0.205, *p*＜0.001). No effect of structural social capital on quality of life was found as a result. Living arrangements played a moderation effect on the relationship between CSC and MCS (*β*=-2.018, *p*＜0.05). The MCS score of high CSC group (55.516 ± 0.757) was significantly higher than that of low CSC group (49.383 ± 0.722).

**Conclusion:**

The results suggest empty-nest elderly has poorer physical health and weaker structural social capital, while the cognitive social capital has a greater positive impact on their quality of life. Targeted interventions to enhance community social capital may be beneficial to improve health status of this vulnerable population.

## Introduction

Empty-nest elderly refers to the elderly who have no children or whose children are absent for a long time, living alone or with their spouse. Due to the lack of children’s company during the individual life cycle transformation, the physical and mental health of empty-nest elderly will be affected and health problems will be more prominent [1–3]. Population ageing is a serious global social problem in the 21st century. It is estimated that by 2050, the global population aged 60 and over will increase from 926 million in 2017 to 2.1 billion, of which 80% are in developing countries [4, 5]. China has the largest elderly population in the world, which is still growing. Seventh National Population Census in China showed that elderly population over the age of 65 had reached 190 million, accounting for 13.5% [6]. With the rapid development of aging in China, the implementation of the former one-child policy and the change of traditional old-age care concepts, the empty-nest phenomenon in China will become increasingly serious, and the number of empty-nest elderly is expected to account for 90% of the country’s elderly population by 2030 [7]. In urban China, the proportion of older adults living alone or only with their spouses is about 50%, rising to over 70% in large cities, which is much higher than those in rural areas (37%) [8]. Therefore, paying attention to the health problems of empty-nest elderly in China is of vital importance to the development of healthy aging in the world.

The elderly have poor self-care ability and need help from their children. As a special group of the elderly, the children of the empty-nest elderly may not be able to provide them with good care support, so they are more likely to get sick and lead to a decline in health [1, 2]. They also face the loneliness caused by living alone or only with their spouse, resulting in a series of negative emotions and psychology, namely “empty nest syndrome” [9]. Many studies confirmed that the empty-nest elderly had worse physical function and were more prone to mental health problems such as depression and anxiety [10]. The factors affecting the health status are complex, and the reasons for their health differences are different. [3, 11, 12]. In order to effectively improve the health status of empty-nest elderly, we need to focus on this population and conduct in-depth analysis of the main influencing factors, so as to put forward targeted suggestions for empty-nest elderly with different characteristics. With the rapid development of modern society, in addition to important sociodemographic factors such as gender, age and education [1–3, 13, 14], the impact of life and social environment on the health of empty-nester elderly is becoming more and more prominent. Among them, Living arrangements is an important factor leading to their health differences. For the empty-nest elderly, the living arrangements is divided into living with their spouse and living alone. According to the research report, compared with people living with their spouses, the anxiety level of those living alone is significantly higher, but there is no significant difference in mental health level between the elderly living with their spouses and the non-empty-nest elderly [2, 12]. It is speculated that the elderly living alone are more vulnerable to the adverse effects of empty-nest than the elderly living with their spouses. However, Ye Chang’s study found no significant difference in health status between the two groups [15]. In recent years, the social determinants of health have received increasing attention. As one of the important factors, social capital has been proved to have an impact on health. It has been proved to be significantly related to self-rated health and obesity, and high social capital may be a protective factor against loneliness and depression [16–18]. At present, more and more practical studies begin to pay attention to the association between social capital and the health of the elderly, and discuss how to promote the health and social adaptability of the elderly from the perspective of social capital [19–21]. However, these studies mainly focus on the health and social capital of the general elderly. Compared with the general elderly, the empty-nest elderly may have worse health conditions, and their family social capital may be more lacking due to the lack of family companionship, which will make them more dependent on other social capital. Therefore, the social capital status of empty-nest elderly and its relationship with health need to be further studied.

The definition of social capital is still controversial. From the perspective of individual social network relations and social status, Bourdieu emphasizes that social capital is an individual’s ability to strengthen society through participation in community activities, and constantly accumulate social resources to obtain more benefits [23]. Putnam proposed from the macro level of democratic governance that social capital is a characteristic of social organizations, such as trust, norms and networks, which can improve social efficiency by promoting cooperative action [22]. Lin Nan proposed from the micro level that social capital is an investment in social relations that expects returns in the market, and is a resource embedded in social networks that actors acquire and use in their actions [24]. Coleman adopted a functionalist definition of social capital. Social capital is defined according to its function. It is not a single entity, but there are many kinds, which have two things in common with each other: they all include some aspects of the social structure and are conducive to the actions of actors in a certain structure, whether individual or collective. Social capital is specifically linked with some activities. Some specific forms of social capital may be useless or even harmful to other activities while promoting some activities [25]. This definition not only explains the difference of individual micro phenomena, but also realizes the transition from micro to macro. Different from Bourdieu, Coleman extends the scope of this concept from the elite group to include social relations of non-elite groups [26]. Patnam understands social capital from the perspective of the impact of social structure on individual behavior and even shaping [27]. It lacks the theory of social capital production, maintenance and growth mechanism [28]. Coleman, on the other hand, explains the micro basis and instrumental function of social capital from the basic analysis method of rational choice theory. Lin nan absolutizes the function of social capital based on whether the function is realized or not, while Coleman proposes an abstract and theoretical function, which is a possibility of realizing the function. In addition, Lin Nan’s research on social capital is limited to the field of social networks [29, 30]. From the above comparative analysis, it can be seen that Coleman’s social capital theory has a broader scope, and its greatest contribution lies in the abstract concept and theoretical enlightenment of social capital. Therefore, this study adopts Coleman’s definition of social capital. A review study demonstrates that social capital is associated with various positive health outcomes [31]. There are many assumptions about the mechanisms by which social capital may improve health, including the dissemination of information and norms on health behavior, the promotion of access to and use of health services, the provision of economic and material resources, and the cultivation of psychological support [32]. However, the association between social capital and health is complex and not absolute [33, 34]Some studies have found that the association between social capital and physical health and the association between community cognitive social capital and self-rated health is not significant [16, 33–35]. In addition, social capital may also have a negative impact on health. Elderly with higher social capital have higher risk of obesity [18]. Social capital may lead to bad health behaviors, such as social drinking, smoking, which may affect health [36, 37]. It may also lead to interpersonal stress and increase additional responsibilities. In social participation and communication, individuals, especially those with high socio-economic status, may bear responsibilities beyond their ability in order to save face, which will exert pressure on individuals and have a negative impact on mental health [38]. Therefore, the role of social capital is still controversial, which may be related to the environment of the study population, or to different dimensions, levels and settings of social capital [39].

On the level and dimension division of social capital, firstly, it can be divided into different levels such as individuals, communities, families, and macro [40]. Although in the digital age, social capital can be obtained beyond geographical boundaries, online resources may not cover all the elderly, and simple online social capital cannot meet the growing needs of the elderly. Therefore, offline social capital may be more important to the elderly. China’s administrative jurisdiction system determines that the community where citizens live is the nearest administrative unit. Affected by their abilities, the activities of the elderly are mostly confined to the communities they live in. Community development is a worldwide movement initiated by the United Nations, which purpose is to strengthen the connection between the national government and the community, give full play to the enthusiasm of community members, make use of the community’s own strength, improve the social and economic development level of the community, improve the life of community residents, and solve the social problems existing in the community. Community is also the focus of China’s social governance. China’s old-age policy advocates “Aging in place”, which means providing medical facilities and activity places around the community where the elderly originally live, so that the elderly can spend their twilight years in a familiar living environment and satisfy their emotional attachment to the long-term living community. Empty-nest elderly may be more reliant on the resources provided by the community in the absence of family resources. Therefore, it is more meaningful to focus the role of social capital on the health of the empty-nest elderly at the community level. As an aspect of social environment, community social capital has been confirmed by some studies to have a significant impact on the health of the elderly [16, 35, 41] [42] [42, 43]. This study takes the empty-nest elderly in the community as the research subject and pays attention to their social networks and resources in the community. According to the theoretical research of social capital, social capital can be further divided into two dimensions: structural social capital (SSC) and cognitive social capital (CSC). Structural social capital refers to relatively objective and externally observable structural resources, such as those obtained in networks and organizations. Community networks and organizations include networks formed by formal citizen participation and networks formed by informal voluntary organization groups and activity participation. In China, the construction of formal organizations is more systematic than that of informal organizations. It is also the duty of every citizen to participate in residents’ meetings and elections. Therefore, formal activity participation is an important way for the elderly to develop community relations. Cognitive social capital mainly describes the perception of non-objective fields, which is manifested in the value beliefs, attitudes and perceptions of relationships within or between groups involving social trust, the principle of reciprocity, social cohesion and social belonging. The community is a collective. The mutual trust, reciprocity and sense of belonging of the residents in the community represent a common understanding of this collective relationship and value. This is to judge the social capital status of community residents from a subjective perspective. [44]. According to previous studies, compared with structural social capital, cognitive social capital seems to have a greater impact on health, but Hu’s research found that structural social capital seems to be more able to prevent cardiovascular disease and death [39, 45].

Uphoff pointed out that not everyone has access to the same sources of social capital and not everyone benefits in the same way [46]. The buffer hypothesis suggests the benefits of social capital to the health of people with a disadvantaged position in society may be greater [46]. Compared with the elderly living with their spouses, the elderly living alone are a more vulnerable group of empty-nest elderly. Some studies have pointed out that the effects of social network size on psychological well-being of people living alone were stronger than that of those living with a spouse [47]. However, there are also hypotheses that vulnerable individuals may miss the beneficial effects of social capital because of social pressure and exclusion [46, 48] [47]. The elderly living alone for a long time may form a closed psychological state, resulting in less social participation and smaller social network, which not only aggravates their psychological loneliness, but also may weaken the health benefits brought by social capital [49–51]. At present, the moderating role of residential status in the relationship between social capital and health is still unclear. Therefore, it is necessary to deeply analyze the relationship between social capital and quality of life of empty-nest elderly by living arrangements.

There are many evaluation indicators about health. With the deepening of people’s understanding of health and the expansion of the concept of health, the concept of quality of life has attracted more and more attention. Quality of life is a comprehensive and complex concept. The World Health Organization defines quality of life as the individual’s perception of his position in life in the context of culture and system values in which he lives and about his goals, expectations, and concerns [52]. With the in-depth development of population aging, the quality of life of the elderly has gradually become the focus of research, but the research development for this group is relatively late. In the early 1980s, United Nations Educational, Scientific and Cultural Organization (UNESCO) began to emphasize the research on the quality of life of vulnerable groups, including the elderly [53]. The Chinese Geriatrics Association defines the quality of life of the elderly as the satisfaction of the elderly aged 60 or over with their physical and mental status, internal and external life of the family [54].The quality of life of the elderly has its particularity as well as commonness compared with the general population. Its particularity is mainly manifested in the healthy life quality of the elderly [55]. Health-related quality of life (HRQOL) is a widely used subjective health evaluation tool, which evaluates individuals’ daily activities, physiological functions, and subjective satisfaction in emotional and social life [56]. HRQOL is described as: “The health aspect of quality of life that focuses on people’s level of ability, daily functioning and ability to experience a fulfilling life” [57]. As HRQOL comprehensively reflects the health-related factors of older adults and covers all aspects of the biopsychosocial model [58], this study uses it for health evaluation.

Since the reform and opening-up, China’s western region, represented by Sichuan Province, has always been a major labor export province. With the acceleration of aging and urbanization, the empty nest problem in the western region is becoming more and more serious. The quality of life of empty-nest elderly is also affected by objective health conditions, environment and other complex factors. Based on the factor of social capital, this study discusses the impact of social capital on the quality of life among empty-nest elderly in Western China from two dimensions (SSC and CSC) at the community level, and discusses the moderating effect of the living arrangement of empty-nest elderly, to provide a reference basis for improving the quality of life of empty-nest elderly.

## Materials and methods

### Data

The data of this study comes from the field survey data of the Youth Science Fund Project of the National Natural Science Foundation of China. The survey is a cross-sectional survey on social capital and health of residents conducted from 2018 to 2019 in Chengdu, a city in Western China. Chengdu, the capital of Sichuan Province, is an important central city in western China. In 2019, the elderly population of Chengdu was 3.1604 million, accounting for 21.07% of the registered population, and the aging rate was higher than the national average (18.13%). According to the 2010 census data provided by the Chengdu Municipal Bureau of Statistics, the proportion of empty-nest elderly in the elderly households in the city has reached about 33%, and the number of single elderly households in the city was 248,000, accounting for 57.9% of empty-nest families. A multi-stage stratified random sampling was used to select the study area. Three urban areas in Chengdu were selected according to the level of economic development and two communities/neighborhood committees were selected from each sample area. Through a systematic random sampling method, the families with odd house number were selected for the questionnaire survey. The sample size is calculated according to the formula n = [µ^2^_α/2_ π (1 - π)]/δ^2^, π = 64.9% (Prevalence of chronic diseases among the elderly in Sichuan Province in the sixth health service survey organized by the National Health Commission in 2018), α = 0.05 and µ^2^_α/2_ = 1.96, and the calculated sample size is 547 people. Considering the situation of no response and deletion, it is expanded by 10% on this basis, and the final sample size is 602. The elderly aged 65 and above were included in this study. According to the question in the questionnaire, “Who do you currently live with?“ If they answered “Living alone” or “Living only with their spouse”, they were regarded as empty-nest elderly and included in this study. Finally, a total of 638 empty-nest elderly completed the interview. The definition criteria of the participants were: (1) aged 65 and above; (2) living alone or with a spouse; (3) living in the community for half a year or more; (4) free of hearing and speaking barriers and voluntary participation. This study was approved by the Ethics Committee of West China School of Public Health and West China Fourth Hospital, Sichuan University, and was in accordance with the Helsinki Declaration of 1964.

### Variables

#### Dependent variables: physical health summary (PCS) and mental health summary (MCS)

The outcome variables of the study were quality life of empty-nest elderly, which was measured by the SF-12 scale due to its scientific and comprehensive nature. The standard SF-12 Health Survey (SF-12 v2), an abbreviated form of the SF-36 that yields the physical health summary (PCS) and mental health summary (MCS) scores, is becoming a popular HRQOL measure in clinical trials because it can be completed in a few minutes [59, 60]. PCS includes the Physical Functioning (PF), Role-Physical (RP), Bodily Pain (BP) and General Health (GH), and MCS includes the Vitality (VT), Social Functioning (SF), Role-Emotional (RE) and Mental Health (MH). The 12 items include two items from each of the PF (Moderate activity and Climb several flights), RP (Accomplished less and Limited in kind), RE (Accomplished less and Did work less carefully) and MH (Felt calm and Felt downhearted) scales and one item from each of the BP (Pain impact), GH (Health in general), VT (Lot of energy) and SF (Social Impact II) scales of the SF-36 [61, 62]. The total score ranged from 0 to 100 for both dimensions, and higher scores indicated better HRQOL. The reliability and validity of the Chinese version of SF-12 in the Chinese population have been widely verified [61]. The Cronbach’s α of PCS in this study is 0.818, and the Cronbach’s α of MCS is 0.835.

#### Independent variables: social capital

The previous research constructed the social capital evaluation index system through the literature method and Delphi expert consultation method, including five levels: individual, family, community, work unit and macro, which has been proved to have good reliability and validity in urban population [33, 63]. Based on the index system, the community social capital questionnaire used in this study was divided into two dimensions: structural social capital and cognitive social capital.

Structural social capital (SSC) was measured by three community networks and participation indicators: (1) “Are you a member of the community organization (political parties / trade unions / voluntary organizations / activity groups, etc.)?” (2) “Have you ever participated in the election of community neighborhood committee (or resident representative)?” (3) “Have you participated in the community residents’ meeting in the last year?” The answer was a binary variable: 0 = No; 1 = Yes. If the response was “yes”, this was scored as 100; If not, this was scored as 0. The score of structural social capital was the average of the three indicators. Higher scores represented higher structural social capital. According to the P-P diagram, the SSC score approximately follows the normal distribution, so taking the average score of SSC score as the boundary. 0 = Low SSC for those less than the average and 1 = High SSC for those greater than or equal to the average. The Cronbach’s alpha was 0.700.

Cognitive social capital (CSC) was measured by five Community trust, belonging and reciprocity indicators: (1) “Do you trust other residents in your community?” (2) “Do you trust the community committee?” (3) “Are you concerned about what happens in your community?” (4) “Do you feel sad if you had to move out of your current community?” (5) “In daily life, do you have reciprocal behavior with the residents of the community?” Each response was measured by 5-point Likert scale: 1 = Strongly disagree, 2 = Disagree, 3 = Not sure, 4 = Agree, and Strongly agree = 5, with scores of 0, 25, 50, 75 and 100 respectively. The score of cognitive social capital was the average of the five indicators. Higher scores represented higher cognitive social capital. According to the P-P diagram, the CSC score approximately follows the normal distribution, so taking the average score of CSC score as the boundary. 0 = low CSC for those less than the average and 1 = High CSC for those greater than or equal to the average. The Cronbach’s alpha was 0.704.

#### Moderator variable: living arrangements

The study mentioned earlier that empty-nest elderly include two categories of elderly people who live alone and those who only live with their spouse, and there may be differences in social capital and health between the two categories. Therefore, this study takes living arrangements as the moderating variable, which was divided into 0 = Living with spouse and 1 = Living alone.

### Covariates

Participants provided information on their sociodemographic characteristics, health status, health-related behavior. Age was measured in years and grouped by 0 = 65–79, 1 = 80 and older. Sociodemographic characteristics include age (0 = 65–79, 1 = 80 and older), gender (0 = Male, 1 = Female), marriage (0 = Married, 1 = Divorce/Separate/Widowed), education level (0 = Not attending primary school, 1 = Primary schools, 2 = Junior high school, 3 = Senior high school, 4 = College school and above) and work status (0 = Never worked, 1 = Retirement/Currently working). Health status included chronic illness (0 = Yes, 1 = No) and two-week illness (0 = Yes, 1 = No). Health-related behavior included: (1) smoking status: 0 = Yes, 1 = Have given up smoking, 2 = No; (2) drinking status: 0 = Yes, 1 = No (Considering the low drinking rate of the respondents, only 12.5%, if the frequency of drinking is subdivided, the sample is small. Therefore, the study classified almost no drinking, drinking only on special occasions such as festivals or holidays, and having abstained from drinking for more than 1 year as no drinking); (3) weekly physical exercise frequency: 0 = Less than 3 times, 1 = 3 times and above; (4) daily sleep time: 0 = Less than 8 h, 1 = 8 h and above. (5) annual physical examination: 0 = No, 1 = Yes.

### Statistical analysis

After quality inspection, Epidata3.1 was used for data input and cleaning, and the Statistical Package for the Social Sciences-23.0 for Windows (SPSS, Inc., Chicago, IL, the United States) was used for statistical analysis. In descriptive statistical analysis, classification variables were described by frequency and composition ratio, while continuous variables were described by mean and standard deviation.Chi square test was used to analyze social capital by living arrangements, and t-test was used to analyze quality of life by living arrangements. The association between social capital, living arrangements and quality of life of empty-nest elderly was analyzed by hierarchical multiple regression analysis. Further, the moderation effect of living state was analyzed by simple effect test. If p < 0.05, it was statistically significant.

## Results

### Descriptive information of the participants

In this study, the average age of participants was 73.61, 19.5% were 80 or older. More than half of the participants were female, 56.9% were married, only 15.0% completed high school or above, and 58.0% never worked. The health of the participants was not good, 59.1% of participants had chronic diseases and 53.0% had an illness within two weeks. Results on health-related behaviors showed that most of the participants did not drink or smoke, exercised three or more times a week and had annual physical examination. However, 64.7% of the participants slept less than 8 h a day on average. Regarding the living arrangement of empty-nest elderly, 66.8% lived with a spouse. (Table 1)

**Table 1:** Sample characteristics (N = 638)

Variable	n (%)
Age	
65–79	520(81.5)
80 or older	118(19.5)
Gender	
Male	191(29.9)
Female	447(70.1)
Marital status	
Married	363(56.9)
Divorce/Separate/Widowed	275(43.1)
Education	
Not attending primary school	132(20.7)
Primary school	274(42.9)
Junior high school	136(21.3)
Senior high school	62(9.7)
College school and above	34(5.3)
Employment	
Currently working/Retirement	268(42.0)
Never worked	370(58.0)
Chronic disease	
Yes	377(59.1)
No	261(40.9)
Two weeks of illness	
Yes	338(53.0)
No	300(47.0)
Drinking	
Yes	80(12.5)
No	558(87.5)
Smoking status	
Yes	72(11.3)
Have given up smoking	50(7.8)
No	516(80.9)
Weekly exercise frequency	
Less than 3 times	106(16.6)
3 times or more	532(83.4)
Sleep time	
Less than 8 h	413(64.7)
8 h or more	225(35.3)
Annual physical examination	
No	251(39.3)
Yes	387(60.7)
Living arrangements	
Living with spouse	426(66.8)
Living alone	212(33.2)

### Quality of life and social capital of empty-nest elderly in different living arrangements

The average scores of PCS, MCS, SSC and CSC of empty-nest elderly were 44.82 ± 11.73, 51.82 ± 11.09, 45.61 ± 38.29, 67.17 ± 15.91 respectively. Stratified by living arrangements, the PCS score in empty-nest elderly living with their spouses was higher than that in the single living group (*p* < 0.05). The differences in SSC and CSC levels were reversed for living arrangements, with significantly more elderly living with a spouse at high levels of SSC than those living alone, yet more elderly living alone were at high levels of CSC. (*p* < 0.05). (Table 2)

**Table 2:** A cross-table showing the health-related quality of life and social capital of empty-nest elderly in different living arrangements

Variables	PCS (M ± SD)	MCS (M ± SD)	SSC (n/%)			CSC (n/%)	
			Low	High		Low	High
Living with spouse	45.66 ± 10.88	52.31 ± 10.90	198 (46.48)	228 (53.52)		223 (52.35)	203 (47.65)
Living alone	43.14 ± 13.16	50.85 ± 11.43	117 (55.19)	95 (44.81)		89 (41.98)	123 (58.02)
χ^2^/t	2.403^*^	1.568	4.296^*^			6.088^*^	

Note: * p < 0.05, ** p < 0.01, *** p < 0.001

### The association between social capital, living arrangements and quality of life of empty-nest elderly

Hierarchical multiple regression analysis was used to analyze the association between social capital, living arrangements and quality of life of empty-nest elderly. Four hierarchical models were constructed with PCS and MCS scores as dependent variables, social capital as independent variable and living arrangements as moderator variable. Model 1 included control variables such as sociodemographic characteristics, health status and health-related behaviors. Model 2 includes independent variables on the basis of model 1. The results showed that after controlling the relevant variables, the CSC of empty-nest elderly had a positive relationship with physical (*β* = 0.188, *p*＜0.001) and mental health (*β* = 0.205, *p*＜0.001). Model 3 further incorporates moderator variable. The results showed that there was still a positive relationship between CSC and PCS and MCS scores, and living arrangements was significantly associated with MCS. In order to test whether living arrangements plays a moderation effect on social capital and quality of life, model 4 further includes the interaction term between social capital and living arrangements. The results showed that there was a significant interaction between CSC and living arrangements on MCS (*β*=−0.079, *p*＜0.05), that is, living arrangements played a moderation effect on the relationship between CSC and MCS (Tables 3 and 4).

**Table 3:** Hierarchical multiple regression of the relationship between social capital, living arrangements and PCS of empty-nest elderly

Variables (Reference)	Model 1			Model 2			Model 3			Model 4	
	β	t		β	t		β	t		β	t
Age (65–79)	-0.115	-2.988^**^		-0.120	-3.172^***^		-0.119	-3.140^**^		-0.118	-3.102^**^
Gender (Male)	-0.056	-1.221		-0.062	-1.369		-0.061	-1.342		-0.066	-1.449
Marital status (Married)	-0.040	-1.023		-0.057	-1.464		-0.019	-0.381		-0.020	-0.394
Education (Not attending primary school)											
Primary school	0.007	0.152		0.016	0.333		0.014	0.297		0.017	0.350
Junior high school	0.057	1.147		0.047	0.952		0.044	0.891		0.052	1.054
High school	0.093	2.057^*^		0.092	2.061^*^		0.091	2.050^*^		0.101	2.258^*^
College school and above	0.067	1.557		0.079	1.886		0.079	1.965		0.079	1.878
Employment (Unemployment)	-0.153	-3.764^***^		-0.119	-2.926^**^		-0.115	-2.834^**^		-0.119	-2.930^**^
Chronic disease (Yes)	0.102	2.100		0.109	2.290^*^		0.111	2.338^*^		0.111	2.335^*^
Two weeks of illness (Yes)	0.180	3.746^**^		0.159	3.373^**^		0.156	3.299^**^		0.157	3.335^***^
Drinking (Yes)	-0.094	-2.410^*^		-0.080	-2.092^*^		-0.080	-2.105^*^		-0.076	-1.973^*^
Smoking status (Yes)											
Have given up smoking	-0.047	-1.027		-0.052	-1.156		-0.052	-1.148		-0.062	-1.371
No	0.060	1.177		0.050	0.991		0.049	0.981		0.045	0.887
Weekly exercise frequency (Less than 3 times)	0.198	5.379 ^***^		0.176	4.799^***^		0.173	4.704^***^		0.176	4.795^***^
Sleep time (Less than 8 h)	0.039	1.063		0.048	1.328		0.048	1.326		0.050	1.406
Annual physical examination (No)	0.004	0.115		0.017	0.460		0.015	0.415		0.015	0.397
SSC (Low)				0.018	0.491		0.015	0.409		0.012	0.310
CSC (Low)				0.188	5.103^***^		0.192	5.193^***^		0.190	5.153^***^
Livingarrangements(Living with spouse)							-0.058	-1.201		-0.059	-1.223
SSC × Living arrangements										0.028	0.778
CSC × Living arrangements										0.069	1.901
R^2^	0.448			0.483			0.485			0.491	
F	9.761^***^			310.491^***^			10.022^***^			9.327^***^	

Note: β = Standardized Coefficient. * p < 0.05, ** p < 0.01, *** p < 0.001

**Table 4:** Hierarchical multiple regression of the relationship between social capital, living arrangements and MCS of empty-nest elderly

Variables	Model 1			Model 2			Model 3			Model 4	
	β	t		β	t		β	t		β	t
Age (65–79)	0.042	1.014		0.041	0.994		0.043	1.054		0.052	1.274
Gender (Male)	-0.029	-0.576		-0.033	-0.668		-0.030	-0.621		-0.028	-0.584
Marital status (Married)	0.000	0.007		-0.021	-0.496		0.053	0.976		0.059	1.082
Education (Not attending primary school)											
Primary school	0.004	0.071		0.011	0.220		0.008	0.155		0.003	0.063
Junior high school	0.138	2.552^**^		0.118	2.212^*^		0.112	2.108^*^		0.107	2.008^*^
High school	0.067	1.367		0.062	1.278		0.060	1.260		0.050	1.032
College school and above	0.081	1.753		0.089	1.957		0.087	1.924		0.090	1.984^*^
Employment (Unemployment)	0.055	1.248		0.096	2.187^*^		0.103	2.342^*^		0.104	2.384^*^
Chronic disease (Yes)	-0.062	-1.179		-0.051	-0.993		-0.047	-0.906		-0.039	-0.757
Two weeks of illness (Yes)	0.114	2.194^**^		0.089	1.747		0.083	1.624		0.079	1.557
Drinking (Yes)	-0.003	-0.081		0.013	0.315		0.012	0.292		0.015	0.353
Smoking status (Yes)											
Have given up smoking	0.009	0.188		0.002	0.042		0.003	0.058		0.014	0.284
No	0.030	0.538		0.017	0.321		0.017	0.304		0.021	0.384
Weekly exercise frequency (Less than 3 times)	0.122	3.056 ^**^		0.091	2.301^*^		0.085	2.149		0.078	1.966^*^
Sleep time (Less than 8 h)	0.129	3.266^**^		0.142	3.652^***^		0.141	3.660^***^		0.143	3.696^***^
Annual physical examination (No)	-0.032	-0.795		-0.016	-0.396		-0.019	-0.477		-0.027	-0.675
SSC (Low)				0.066	1.638		0.060	1.491		0.064	1.596
CSC (Low)				0.205	5.158^***^		0.213	5.349^***^		0.212	5.330^***^
Living arrangements(Living with spouse)							-0.112	-2.163*		-0.109	-2.096^*^
SSC × Living arrangements										0.055	1.397
CSC × Living arrangements										-0.079	-2.018^*^
R^2^	0.246			0.322			0.333			0.344	
F	2.501^**^			3.986^***^			4.045^***^			3.939^***^	

Note: β = Standardized Coefficient. * p < 0.05, ** p < 0.01, *** p < 0.001

### The moderating role of living arrangements in the relationship between cognitive social capital and mental health

**Fig. 1:**
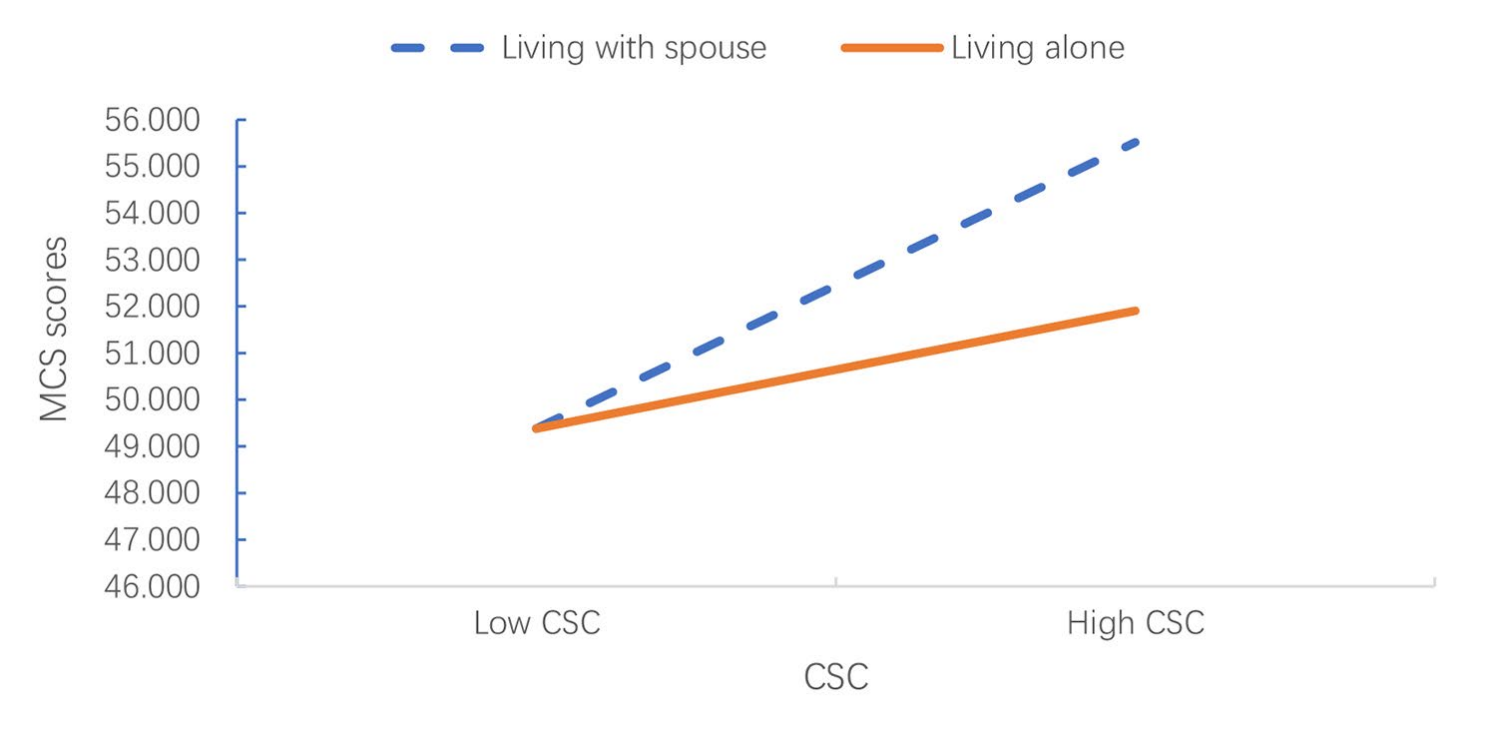
the moderation effect of living arrangement on the relationship between CSC and MCS

Further, the moderation trend of living arrangements on the relationship between CSC and MCS score was analyzed by simple effect test. Results as shown in Fig. 1, for empty-nest elderly living with their spouses, the MCS score in high CSC group was significantly higher than low CSC (55.516 ± 0.757 vs. 49.383 ± 0.722, *F* = 34.385, *p* < 0.001). For empty-nest elderly living alone, there was no significant difference in the MCS score between the two groups (*F* = 2.834, *p* > 0.05).

## Discussion

The purpose of this study is to explore the relationship between community social capital and the quality of life of empty-nest elderly and the moderation effect of living arrangements. It was found that there were differences in the social capital level of empty-nest elderly with different living arrangements, and there were differences in physical health of empty-nest elderly with different living arrangements. Cognitive social capital has a positive impact on the quality of life for empty-nest elderly, and structural social capital has no significant impact on the quality of life. Living arrangements moderates the relationship between cognitive social capital and mental health.

### The physical health of empty-nest elderly living alone is worse

The empty-nest elderly is vulnerable to different disadvantageous situations and experience problems associated with old age, such as physical and mental health problems [15]. Previous studies have confirmed that the health status of empty-nest elderly is worse than that of non-empty-nest elderly [12, 64, 65], but the health differences among different groups of empty-nest elderly are not clear. This study found that there was heterogeneity in the quality of life of the urban empty-nest elderly with different living arrangements, that is, the PCS scores of the elderly living alone were significantly lower than those living with their spouses. This may be because the empty-nest elderly living with their spouses are more conducive to social support exchanges, healthy lifestyles, and economic resources, which can better protect the health of them [11, 66, 67].There was no significant difference in mental health among empty-nest elderly of different living, which may be because no matter which group of empty-nest elderly, they lack family companionship. Their mental health is generally not very good, which may be more affected by other social factors [7, 65, 68].

### Structural social capital of empty-nest elderly living with their spouses was higher, but cognitive social capital of those living alone was higher

Social capital is an invisible health resource, which has an important impact on people’s health. At present, China’s social security system for the elderly is still underdeveloped. In addition, influences by Chinese traditional thought, Chinese elderly tend to establish relationships through geographical ties and kinship in order to achieve similar functions and collective interests [69]. Based on lack of kinship, empty-nest elderly may rely more on the social resources provided by geographical relations, which mainly comes from the community. Structural social capital describes the networks, relationships, and institutions that link people and groups together, while cognitive dimension reflects the values, trust, confidence, and norms that characterize these relationships [44]. In this study, there were significant differences in structural social capital among groups of different living arrangements. The elderly living with their spouses have higher structural social capital, but lower cognitive social capital. This may be explained by the fact that elderly living alone lack the company of their spouses and children, will have more contact with community residents, and therefore they will trust and rely on them more. However, since they need to take care of themselves, they may not have the energy to participate in community activities and affairs.

### The structural social capital of the empty-nest elderly is weak, and it has no significant impact on the quality of life

As for the impact of social capital on quality of life, this study did not find any effect of structural social capital. Previous studies have also proposed that cognitive social capital will have a greater impact on health than structural social capital [70], but in this study, it may be because the structural social capital of the study population is generally weak. It was found that the average score of structural social capital of empty-nest elderly was only 45.61 ± 38.29, far lower than cognitive social capital (67.17 ± 15.91). It may be precisely because the empty-nest elderly have low participation in community organizations and affairs, their structural social capital is insufficient, and is not enough to have an impact on health[69]. However, some studies have found that structural social capital can improve the physical function and mental health of empty-nest elderly [7, 58]. Social participation can increase the activities of the elderly to maintain the physical function, increase their frequency of using local medical services and taking health behaviors to promote their physical health, and also increase their social support and identity to alleviate their depression [7, 16]. It can be seen that the factor limiting the health of the empty-nest elderly may be the lack of structural social capital. Therefore, it is of great importance to improve the structural social capital of the empty-nest elderly.

### Cognitive social capital has a positive impact on the quality of life of the empty-nest elderly

Social capital influences health primarily through the following ways: spreading health promotion knowledge, the maintenance of health behavior, accessing medical and health care services and facilities, accessing emotional or material support, and maintaining mutual respect in social networks [71]. In this study, cognitive social capital had a positive impact on the physical health of empty-nest elderly. The elderly with high cognitive social capital has a stronger sense of trust and belonging to the community, and will make more use of the community’s health and fitness resources, so as to promote their physical health [69]. Cognitive social capital had a positive impact on the mental health of empty-nest elderly. The sense of belonging and security may also have a positive impact on the neuroendocrine status of the elderly [16], which is conducive to their mental health development. At the same time, the empty-nest elderly with high cognitive social capital receives more psychological support in the process of communicating with their neighbors in the community [69].

### Living arrangement plays a moderating role in the relationship between cognitive social capital and mental health

This study finds that the living arrangements of empty-nest elderly can moderate the relationship between cognitive social capital and mental health. This reflects that the effect of social capital may be affected by the environment. The results show that the positive correlation between cognitive social capital and mental health is statistically significant only in the living with spouse group, but not in the living alone group. This may be because the group of living alone belongs to the vulnerable group of the empty-nest elderly with a stronger spiritual emptiness [1, 72]. According to the results of regression analysis, there is a correlation between living arrangements and the mental health of empty nesters. The psychological status of empty-nest elderly living alone is worse than that of those living with their spouses. Therefore, the compensation effect of the community may be small, and they need the company of their families more.

## Conclusion

This study confirmed that community social capital had a positive impact on health-related quality of life of empty-nest elderly, but it was limited to cognitive social capital, and the role of structural social capital was not reflected. After stratified analysis of living arrangements, we found that the elderly living alone, as vulnerable groups in the empty-nest elderly, had lower structural social capital, but had higher cognitive social capital. Living arrangements plays a moderating role in the relationship between cognitive social capital and mental health. The positive relationship between cognitive social capital and mental health is only significant in the elderly group living with their spouses. In addition, educational level, chronic disease, two-week illness and weekly exercise will also affect their health-related quality of life. From the results, it can be inferred that the structural social capital of the empty-nest elderly, especially the elderly living alone, is lacking and does not play a role in health. Therefore, it is necessary to strengthen the construction of community residents’ relationship network, carry out various community activities such as fitness exercise, and encourage the empty-nest elderly, especially the elderly living alone, to actively participate. The community should support them to participate in community affairs in the form of community residents’ assembly. Moreover, the community should pay attention to the establishment of cognitive social capital of empty-nest elderly living with their spouses, because it seems that social capital has a greater impact on the quality of life of this group. Strengthening visits to them, cultivating and developing community social organizations and volunteer teams for them, and actively carrying out community activities during holidays will help to improve their community trust and sense of community belonging.

This study has four limitations that need to be considered. Firstly, this study is a cross-sectional study, which cannot draw the causal relationship between social capital and health-related quality of life of empty-nest elderly, and the health-related quality of life will in turn affect the social capital. Therefore, it needs to be further verified by prospective research. Secondly, this study is a retrospective study of the elderly, there may be some memory bias, which will affect the analysis results. Third, this study did not control for the presence of children and other family members living in nearby or the same city, which may have an impact on the results. Finally, the participants of this study are the urban empty-nest elderly in Chengdu. Due to the limitation of sample size, its generalization is not high, and the sample data results of other cities are needed.

## Data Availability

As the data involves privacy and future research, the data sets generated and / or analyzed during this study are not public, but can be obtained from the corresponding authors upon reasonable request.

## Abbreviations

UNESCO: United Nations Educational
PCS: Physical Health Summary
MCS: Mental Health Summary
HRQOL: Health-Related Quality Of Life
PF: Physical Functioning
RP: Role-Physical
BP: Bodily Pain
GH: General Health
VT: Vitality
SF: Social Functioning
RE: Role-Emotional
MH: Mental Health
SSC: Structural Social Capital
CSC: Cognitive Social Capital
